# Ultrasonography of thyroid nodules: a pictorial review

**DOI:** 10.1007/s13244-015-0446-5

**Published:** 2015-11-26

**Authors:** Cheng Xie, Peter Cox, Nia Taylor, Sarah LaPorte

**Affiliations:** Department of Radiology, John Radcliffe Hospital, Oxford University Hospitals Foundation Trust, Headley Way, Headington, Oxford, OX3 9DU UK; Department of Radiology, Churchill Hospital, Oxford University Hospitals Foundation Trust, Old Road, Headington, Oxford, OX3 7LE UK; Department of Radiology, Milton Keynes Hospital, Milton Keynes Hospital NHS Foundation Trust, Standing Way, Eaglestone, Milton Keynes, MK6 5LD UK

**Keywords:** Thyroid nodule, Thyroid cancer, Ultrasound, Fine-needle aspiration, Sonographic features

## Abstract

**Abstract:**

Thyroid nodules are a common occurrence in the general population, and these incidental thyroid nodules are often referred for ultrasound (US) evaluation. US provides a safe and fast method of examination. It is sensitive for the detection of thyroid nodules, and suspicious features can be used to guide further investigation/management decisions. However, given the financial burden on the health service and unnecessary anxiety for patients, it is unrealistic to biopsy every thyroid nodule to confirm diagnosis. The British Thyroid Association (BTA) has recently produced a US classification (U1–U5) of thyroid nodules to facilitate the decision-making process regarding the need to perform fine-needle aspiration cytology (FNAC) for suspicious cases. In this pictorial review, we provide a complete series of sonographic images to illustrate benign and malignant features of thyroid nodules according to the U1–5 classification. Specifically, we highlight morphologic characteristic of the nodule, including its echo signal in relation to its consistency, nodular size, number and contour. Additional diagnostic features such as halo, colloid, calcification and vascular patterns are also discussed in detail. The aim is to assist radiologists and clinicians in recognising sonographic patterns of benign, suspicious and malignant nodules based on U1–5 criteria, and in planning for further investigations.

***Main messages*:**

• *Ultrasound is sensitive in identifying suspicious features, which require aspiration.*

• *Whether nodules require aspiration should be based on sonographic features and clinical findings.*

• *U1–5 classification of sonographic findings can help determine whether aspiration is necessary.*

## Introduction

**Fig. 1:**
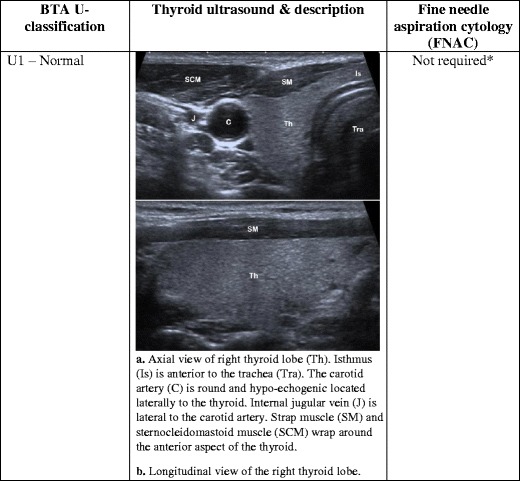
(**a**) Axial view of right thyroid lobe (Th). Isthmus (Is) is anterior to the trachea (Tra). The carotid artery (C) is round and hypo-echogenic located laterally to the thyroid. The internal jugular vein (J) is lateral to the carotid artery. The strap muscle (SM) and sternocleidomastoid muscle (SCM) wrap around the anterior aspect of the thyroid. (**b**) Longitudinal view of the right thyroid lobe

**Fig. 2:**
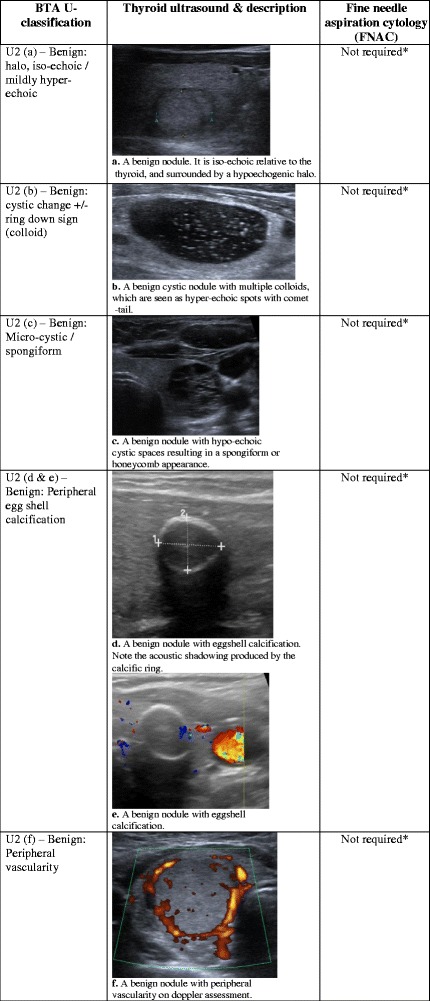
(**a**) A benign nodule. It is iso-echoic relative to the thyroid, and surrounded by a hypo-echogenic halo. (**b**) A benign cystic nodule with multiple colloids, which are seen as hyper-echoic spots with comet-tail. (**c**) A benign nodule with hypo-echoic cystic spaces resulting in a spongiform or honeycomb appearance. (**d**) A benign nodule with eggshell calcification. Note the acoustic shadowing produced by the calcific ring. (**e**) A benign nodule with eggshell calcification. (**f**) A benign nodule with peripheral vascularity on Doppler assessment

**Fig. 3:**
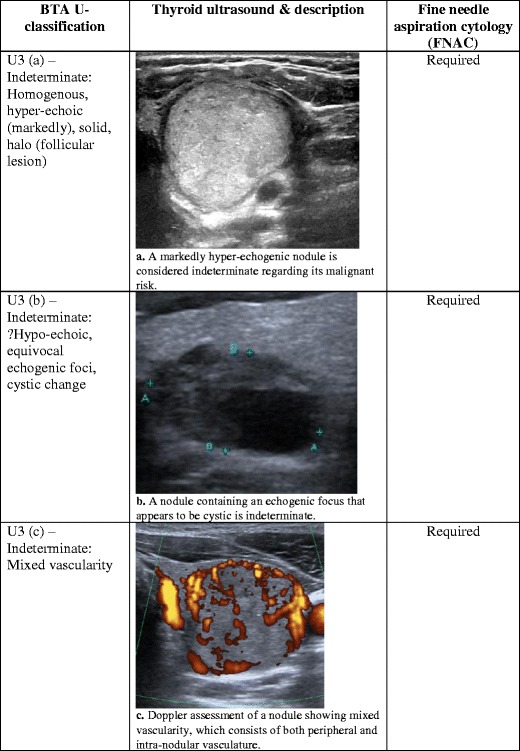
(**a**) A markedly hyper-echogenic nodule is considered indeterminate regarding its risk of malignancy. (**b**) A nodule containing an echogenic focus that appears to be cystic is indeterminate. (**c**) Doppler assessment of a nodule showing mixed vascularity, which consists of both peripheral and intra-nodular vasculature

**Fig. 4:**
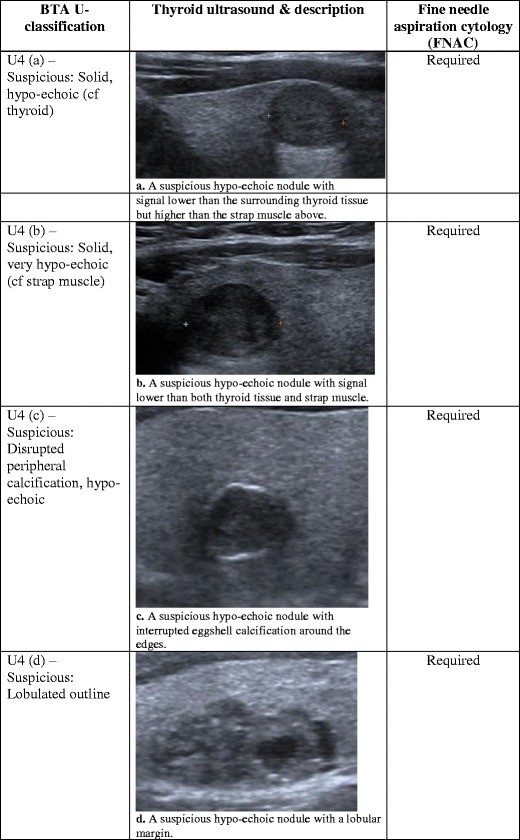
(**a**) A suspicious hypo-echoic nodule with signal lower than the surrounding thyroid tissue but higher than the strap muscle above. (**b**) A suspicious hypo-echoic nodule with signal lower than both thyroid tissue and strap muscle. (**c**) A suspicious hypo-echoic nodule with interrupted eggshell calcification around the edges. (**d**) A suspicious hypo-echoic nodule with a lobular margin

**Fig. 5:**
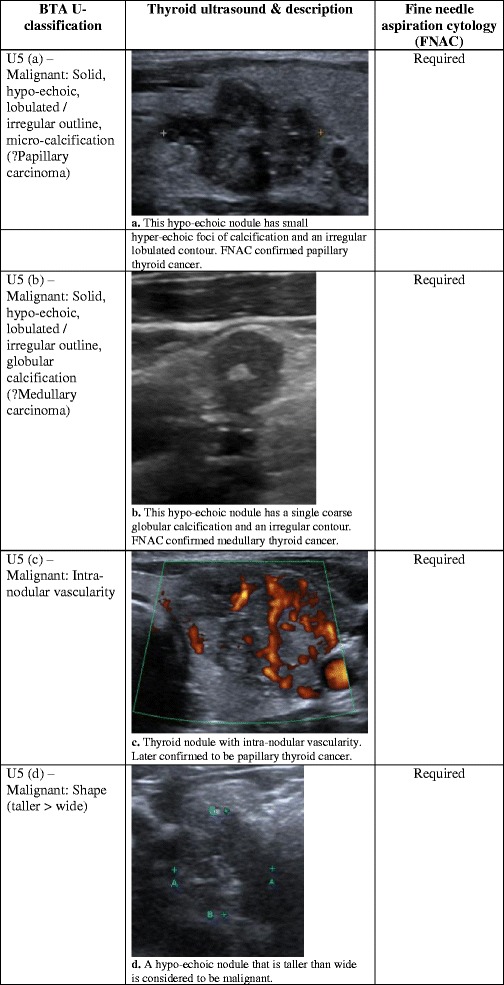

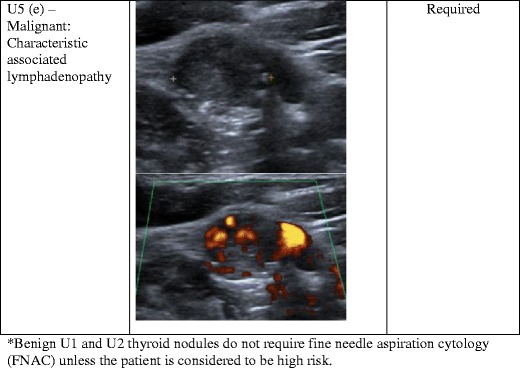
(**a**) This hypo-echoic nodule has small hyper-echoic foci of calcification and an irregular lobulated contour. FNAC confirmed papillary thyroid cancer. (**b**) This hypo-echoic nodule has a single coarse globular calcification and an irregular contour. FNAC confirmed medullary thyroid cancer. (**c**) Thyroid nodule with intra-nodular vascularity, later confirmed to be papillary thyroid cancer. (**d**) A hypo-echoic nodule that is taller than wide is considered to be malignant. (**e**) An abnormal lymph node with malignant features—irregular contours, mixed echotexture and vascularity. An abnormal lymph node would result in a U5 category despite any benign features of the thyroid nodule

A thyroid nodule is a discrete lesion within the normal thyroid. Such nodules are a common occurrence in the general population and a frequent incidental finding on computed tomography (CT) and magnetic resonance imaging (MRI). Autopsy studies have reported incidental thyroid nodules in up to 50% of subjects [1]. Most nodules are benign, but between 3 and 7% of cases are found to be malignant [2]. Ultrasound (US) has become an important diagnostic tool in the assessment of thyroid nodules. It is highly sensitive for detecting nodules, and the sonographic features of the nodules can be used to determine the need for further investigation [3]. A number of studies have investigated both benign and malignant sonographic features of thyroid nodules [1, 4–7]. However, because it is unrealistic from an economic and patient anxiety point of view to biopsy every thyroid nodule in order to exclude malignancy, a reliable guideline was necessary to specifically target nodules that require biopsy. Based on current evidence, the British Thyroid Association (BTA) recently produced a US classification (U1–U5) of thyroid nodules to facilitate the decision-making process regarding the need to perform fine-needle aspiration cytology (FNAC) in suspicious/unequivocal cases [8]. The aim of this pictorial review is to present detailed sonographic images that correspond to each feature as described in the BTA guideline in order to help radiologists and clinicians readily recognise the sonographic patterns and classify nodules into categories of U1 to U5 (Figs. 1, 2, 3, 4, and 5).

## Ultrasound classification (U1): the normal thyroid

US is a safe, fast and comfortable method for evaluating the thyroid gland and regional anatomy. A high-resolution probe between 10 and 15 MHz should be used to examine the neck. The patient should be lying supine and the neck in a slightly hyper-extended position to fully expose the anterior neck. A semi-erect position is acceptable if the patient is unable to tolerate the preferred posture. Before anyone can become adept in identifying abnormalities, it is critical to become familiar with the normal sonographic appearance of the thyroid gland relative to surrounding structures.

The normal thyroid gland is homogenous and is mildly hyper-echogenic compared to surrounding muscles (Fig. 1). Each lobe of the thyroid has a globular shape with a smooth outline. Individual lobes and any suspicious structure must be examined in both the axial and longitudinal views. The isthmus is located at the anterior aspect between the lobes and anterior to the trachea. Two important features make the trachea readily identifiable: the trachea has no US signal, as it is filled with air, which does not transmit US, and the cartilaginous rings at the anterior aspect of the trachea produce curvilinear hyper-echogenic signals. The carotid arteries are round hypo-echogenic tubular structures located laterally to each lobe of the thyroid. Further lateral to the carotid arteries is the paired internal jugular vein, which is often collapsed but can be distended with the Valsalva manoeuvre to aid in identification. Apart from these two large vessels, there are often echo-free thinner vessels travelling within and around the periphery of the thyroid gland. All vascular structures should be closely interrogated with colour or power Doppler imaging. Firstly, this is a useful technique for separating blood vessels from cystic structures. Secondly, the vascular pattern at and around a thyroid nodule is a useful feature in differentiating between benign and malignant lesions. The muscular structures (strap muscle and sternocleidomastoid muscle) wrap around the anterior aspect of the thyroid gland and have lower echo signal relative to the thyroid. Other commonly identifiable regional structures include the lymph nodes, nerves and the oesophagus, which is situated posteromedially to the left lobe of the thyroid. The parathyroid glands are rarely seen unless there is enlargement of the glands.

For anyone performing US examination of the neck, a sound knowledge of the normal anatomy and a systemic scanning approach are prerequisites for confidently identifying and fully characterising thyroid nodules.

## Ultrasound classification (U2): benign thyroid nodule

On US, thyroid nodules are depicted as discrete lesions, as they cause distortion of the homogeneous echo pattern of the thyroid gland [9]. There are various characteristics on US that help to distinguish benign from malignant nodules. However, it is important to emphasise that the size and number of nodules are not reliable factors for disease differentiation. Evidence has shown that nodular size is not predictive of malignancy risk, and that nodular morphology, rather, is the more important criterion [4, 7, 10–13]. In addition, the number of nodules is not associated with higher risk of cancer. Regardless of nodule quantity, the overall incidence of thyroid cancer is shown to be consistently between 9.2 and 13% after FNAC [1, 5, 14].

The following morphologic characteristics are suggestive of a benign thyroid nodule. For signal intensity, the nodule should have the same echo signal or be slightly hyper-echogenic relative to the surrounding normal thyroid tissue. This solid nodule may be accompanied by a hypo-echogenic halo (Fig. 2a), which represents fibrous connective tissue, compressed thyroid tissue and vessels [8]. In cases where the nodule appears cystic, it should contain colloids, which appear as a hyper-echogenic spot with ‘comet-tail’ shadowing or a ‘ring-down’ sign (Fig. 2b) [8]. In addition, some nodules can have a spongiform/honeycomb consistency with dark micro-cystic spaces (Fig. 2c). The spongiform appearance has been shown to have specificity of 99.7% for benign disease and 98.5% negative predictive value for malignancy [7, 9]. At the peripheries of the nodule, complete eggshell-type calcification (Fig. 2d and e) [15, 16] and peripheral vascularity on Doppler assessment (Fig. 2f) are also reassuring signs [7, 17]. A thyroid nodule with these features would be a benign nodule and does not require FNAC. If the patient is asymptomatic and there is no clinical suspicion or risk factors for malignancy, further US follow-up is not recommended by the BTA [8]. Clinical and risk factors that should be considered include [8, 18]:

- Age <20 or > 60 years
- The nodule is firm on palpation
- A history of fast-growing nodule
- Vocal cord paralysis, which should be further investigated
- Regional lymphadenopathy
- Previous radiotherapy to the neck region
- Family history of thyroid cancer

Patients presenting for US assessment of a possible thyroid nodule usually involve cases of an incidental nodule found on other imaging investigations or general practitioner (GP) referrals for a palpable thyroid lump. The majority of nodules will be benign [19], and evidence indicates that incidental nodules, palpable and non-palpable nodules have the same incidence of thyroid cancer [4, 20]. The incidence of thyroid cancer is on the rise, but early detection has not led to any changes in mortality. Studies suggest that the high sensitivity of US has been responsible for the detection of incidental nodules, which has resulted in overdiagnosis of these subclinical cases [21–23]. This evidence supports the opinion that incidental nodules do not require US assessment unless the patient also has risk factors and clinical findings that are suspicious for malignancy [14, 19, 24].

## Ultrasound classification (U3): indeterminate/equivocal

On assessment of the thyroid nodule, there are situations in which, among a collection of benign sonographic appearances, there are certain features that place doubt on the diagnosis. Occasionally, the nodule may be markedly hyper-echogenic (Fig. 3a), or part of the nodule may appear cystic and contain echogenic foci (Fig. 3b). Further Doppler assessment of the nodule may demonstrate a mixture of both peripheral and intra-nodular vascularity (Fig. 3c). These sonographic features are classified as indeterminate. FNAC should be performed, taking into consideration the clinical information [8].

## Ultrasound classification (U4): suspicious thyroid nodule

Thyroid nodules in this category are considered to be suspicious for malignancy, and all these nodules should be further investigated with FNAC [8]. The first distinctive feature of these suspicious nodules is their hypo-echogenicity [8]. The echo signals of the nodule or part of the nodule are less than the surrounding normal thyroid tissue (Fig. 4a) and sometimes lower than the nearby muscle (Fig. 4b). It is important to note that these nodules are hypo-echogenic, but they are also predominantly solid in consistency. This property makes their echo signals higher than those of a cystic nodule, which is dark and echo-free. On a spectrum from highest to lowest likelihood of malignancy, predominantly solid nodules have the highest risk, while mixed solid/cystic sit in the middle, and cystic or spongiform have the lowest risk [1]. Furthermore, the suspicious nodule may have disrupted eggshell calcification around the peripheries (Fig. 4c) or lost its smooth round contour, and adopted a lobulated margin (Fig. 4d) [8]. A U4 thyroid nodule is hypo-echogenic, with an irregular outline and possible disrupted calcification at the edges.

## Ultrasound classification (U5): malignant thyroid nodule

When assessing a thyroid nodule, it is important to note that malignant lesions are rare. The incidence of the disease is 2–4 cases per 100,000 persons per year [25]. The most prevalent form of thyroid cancer is papillary thyroid cancer (75-80%), followed by follicular (10-20%), medullary (3-5%) and anaplastic (1-2%) thyroid cancers [2, 26]. The survival rate for thyroid cancer in general is better than for other forms of cancer. For papillary thyroid cancer, the 20-year survival after surgery is around 99% [27].

There are a number of features on US that are suggestive of a malignant thyroid nodule. Such nodules are frequently hypo-echoic on US relative to the adjacent normal thyroid tissue [8]. However, this is not diagnostic, and should be considered with other features indicative of malignancy. On measuring the size of the nodule, it is necessary to assess both the anteroposterior (AP) and transverse (TR) diameters in the axial plane. When the AP diameter is more than the TR diameter, the nodule is described as ‘taller than wide’ in shape, which increases the risk of malignancy (Fig. 5d) [28]. The additional presence of hyper-echoic foci of calcifications within the nodule further increases the likelihood of cancer—approximately three times the cancer risk for micro-calcifications and twice the risk for coarse calcifications [1]. Micro-calcifications have been reported to have specificity of 44–95% for thyroid cancer, and are particularly associated with papillary thyroid cancer (Fig. 5a). However, the sensitivity of micro-calcifications is low (26–59%) [1, 7, 29, 30]. Coarse macro-calcifications are not specific for malignancy, as they commonly occur in multinodular goiters. However, in situations where there is a single nodule containing coarse calcification, the risk of cancer can be as high as 75% [31]. Coarse calcification is often associated with medullary thyroid carcinoma (Fig. 5b). A malignant nodule can also have an irregular shape with irregular edges (Fig. 5a). Further evaluation of the nodular vascularity should be performed with colour or power Doppler. Malignant lesions tend to demonstrate intra-nodular vascularity (Fig. 5c). In contrast, benign nodules have a peripheral rim of blood flow (Fig. 2f) [1]. When both intra-nodular and peripheral vascularity are present, the nodule is indeterminate (Fig. 3c). All U5 nodules should be investigated with FNAC.

**Fig. 6:**
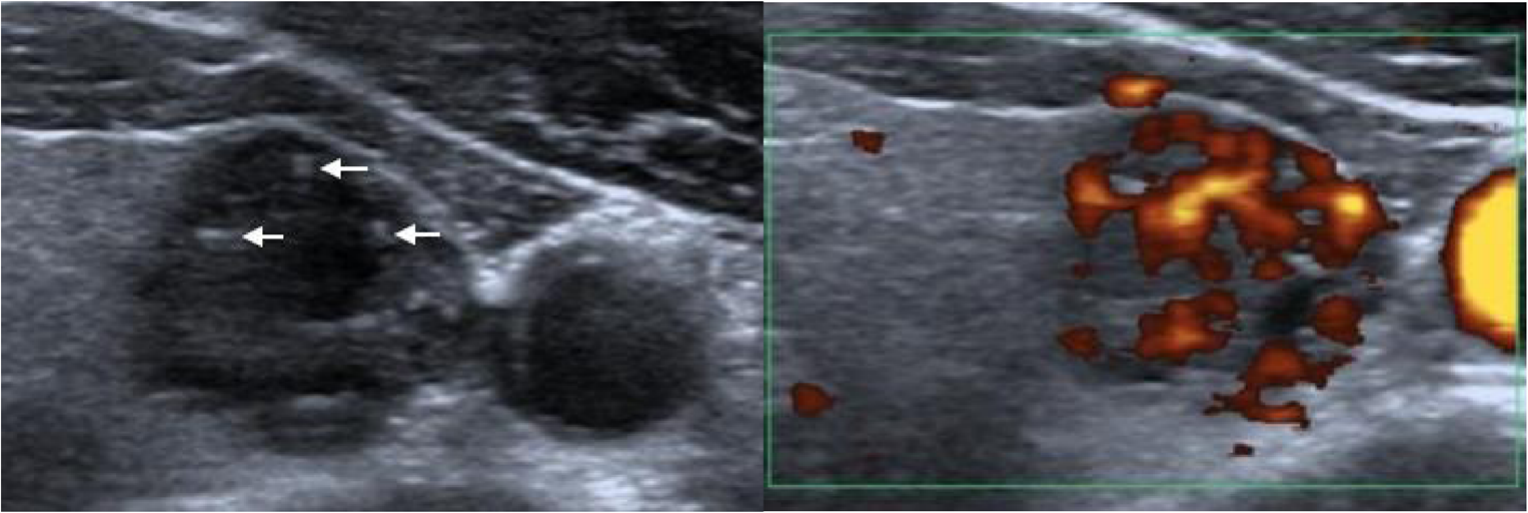
The thyroid nodule in the left lobe consists of colloids with their characteristic ‘comet-tail’ shadowing (arrows). However, it is hypo-echoic with an echo signal close to the adjacent strap muscle. Power Doppler shows marked intra-nodular vascularity. Multiple sinister characteristics of the nodule put it between U4 and U5, which require FNAC

It is important to emphasise that no single sonographic feature should be used to differentiate between benign and malignant nodules [13]. Instead, the overall appearance and collective features of the thyroid nodule should be considered for diagnosis. Figure 6 illustrates a nodule in the left lobe of the thyroid. It consists of colloids, with their characteristic ‘comet-tail’ shadowing. Colloids, as discussed earlier, are features indicative of benign nodules. However, the nodule is hypo-echoic with an echo signal close to the adjacent strap muscle. In addition, the posterior aspect of the nodule appears irregular and lobulated. Power Doppler shows marked intra-nodular vascularity. For this nodule, there is a benign feature (colloids), but the series of sinister characteristics of the nodule puts it between the suspicious U4 and malignant U5 categories, which require FNAC. This case also illustrates that the presence of malignant features takes precedence over any benign characteristics of the nodule. The results of FNAC of this nodule confirmed papillary thyroid cancer.

## Lymph node assessment

Lymph node involvement is most common in papillary thyroid cancer. In 20% of patients with papillary thyroid cancer, a symptomatic cervical lymph node is one of the main complaints. In addition, nodal metastases have been reported in 50% of patients with papillary thyroid cancer on first presentation [32]. In follicular thyroid cancer, metastases have a tendency to spread to distant organs. For medullary thyroid cancer, both cervical lymph nodes and distant organs can be involved [33].

Cervical lymph node assessment should be an integral part of the thyroid nodule examination. The neck should be thoroughly screened for any abnormal lymph nodes. Normal and reactive lymph nodes are usually oval with an echogenic hilum, and they are typically hypo-echoic relative to nearby muscle. On Doppler evaluation, there is normally colour flow at the hilum of a reactive lymph node, but it can also appear avascular [34]. An abnormal lymph node would have lost one or a combination of these normal features in terms of shape, echotexture, fatty hilum, irregular contour and vascularity (Fig. 5e). The presence of an abnormal lymph node would result in a category of U5 regardless of any benign features of the thyroid nodule. Abnormal lymph nodes should undergo FNAC along with the nodule to facilitate accurate diagnosis and staging [8].

A thorough discussion of cervical lymph node evaluation is beyond the scope of this article. However, a number of studies have illustrated in detail the sonographic characteristics of neck lymph nodes [34–37], which the reader should find useful for guiding the ultrasound examination.

## Reporting findings

In providing a report after the US examination of the thyroid nodule, the practitioner should aim to address several factors regarding the nodule. The report should include a detailed description of the nodule with regard to its size, location, composition and echogenicity in relation to surrounding structures. The nodular margins should be closely examined to indicate the contour and presence of rim calcification or halo, and any additional features such as colloid calcification and vascular pattern. The lymph nodes should also be screened and commented on if there are any suspicious features. Based on the collection of sonographic findings, the practitioner should provide a US classification of the thyroid nodule from U1 to U5. When there are multiple nodules, the most suspicious nodule should be used for scoring [8]. If FNAC was performed, this should also be clearly documented on the report. The clinical team should be informed of the results, irrespective of benign or malignant disease. A multi-disciplinary approach is recommended if malignancy is suspected or when there is discordance between US or FNAC findings and clinical evidence [8].

The BTA thyroid nodule U-classification is designed to simplify the process of identifying suspicious and malignant nodules. Its purpose is to provide guidance on the appropriate use of FNAC. Benign thyroid conditions, such as thyroiditis, are not part of the U-classification, and are not included in the pictorial review. Apart from the BTA U-classification, there are other recognised guidelines for the management of thyroid nodules [9, 11, 38]. A comparison of the U-classification with other guidelines is beyond the scope of this pictorial review, which serves to illustrate the sonographic features as described in the BTA guideline. These are limitations of this study.

## Conclusions

US provides a safe and fast method for the examination of thyroid nodules. Such nodules are common, however, and it would be impractical to biopsy every nodule to confirm diagnosis. In this pictorial review, we have provided detailed descriptions with corresponding images of sonographic features of thyroid nodules according to the recent BTA U1–5 classification. We trust that the ready recognition of benign, suspicious and malignant sonographic features based on the U1–5 classification will assist radiologists and clinicians in the subsequent management of thyroid nodules.
